# Deep learning for fully-automated nuclear pleomorphism scoring in breast cancer

**DOI:** 10.1038/s41523-022-00488-w

**Published:** 2022-11-08

**Authors:** Caner Mercan, Maschenka Balkenhol, Roberto Salgado, Mark Sherman, Philippe Vielh, Willem Vreuls, António Polónia, Hugo M. Horlings, Wilko Weichert, Jodi M. Carter, Peter Bult, Matthias Christgen, Carsten Denkert, Koen van de Vijver, John-Melle Bokhorst, Jeroen van der Laak, Francesco Ciompi

**Affiliations:** 1 Radboud University Medical Center, Department of Pathology, Nijmegen, The Netherlands; 2 GZA-ZNA Hospitals, Department of Pathology, Antwerp, Belgium; 3 Peter Mac Callum Cancer Centre, Division of Research, Melbourne, Australia; 4 Mayo Clinic, Department of Laboratory Medicine and Pathology, Rochester, MN USA; 5 Medipath & American Hospital of Paris, Paris, France; 6 Canisius Wilhelmina Ziekenhuis, Nijmegen, The Netherlands; 7 University of Porto, Institute of Molecular Pathology and Immunology Department of Pathology, Ipatimup Diagnostics, Porto, Portugal; 8 The Netherlands Cancer Institute, Department of Molecular Pathology, Amsterdam, The Netherlands; 9 Technical University Munich, Institute of Pathology, Munich, Germany; 10 Department of Laboratory Medicine and Pathology, University of Alberta, Edmonton, Canada; 11 Hannover Medical School, Institute of Pathology, Hannover, Germany; 12 Philipps University of Marburg, Institute of Pathology, Marburg, Germany; 13 Ghent University Hospital and Cancer Research Institute Ghent, Department of Pathology, Ghent, Belgium; 14 Center for Medical Image Science and Visualization, Linköping University, Linköping, Sweden

**Keywords:** Breast cancer, Translational research, Pathology

## Abstract

To guide the choice of treatment, every new breast cancer is assessed for aggressiveness (i.e., graded) by an experienced histopathologist. Typically, this tumor grade consists of three components, one of which is the nuclear pleomorphism score (the extent of abnormalities in the overall appearance of tumor nuclei). The degree of nuclear pleomorphism is subjectively classified from 1 to 3, where a score of 1 most closely resembles epithelial cells of normal breast epithelium and 3 shows the greatest abnormalities. Establishing numerical criteria for grading nuclear pleomorphism is challenging, and inter-observer agreement is poor. Therefore, we studied the use of deep learning to develop fully automated nuclear pleomorphism scoring in breast cancer. The reference standard used for training the algorithm consisted of the collective knowledge of an international panel of 10 pathologists on a curated set of regions of interest covering the entire spectrum of tumor morphology in breast cancer. To fully exploit the information provided by the pathologists, a first-of-its-kind deep regression model was trained to yield a continuous scoring rather than limiting the pleomorphism scoring to the standard three-tiered system. Our approach preserves the continuum of nuclear pleomorphism without necessitating a large data set with explicit annotations of tumor nuclei. Once translated to the traditional system, our approach achieves top pathologist-level performance in multiple experiments on regions of interest and whole-slide images, compared to a panel of 10 and 4 pathologists, respectively.

## Introduction

To guide management, most pathologists grade breast cancers according to a standardized grading system^1,2^, comprised of three features: (1) degree of nuclear pleomorphism (or “atypia”), (2) extent of gland formation and (3) mitotic count. The scoring criteria for mitotic count and gland formation are defined by quantitative measures, whereas nuclear pleomorphism scoring is based on qualitative analysis of the nuclear morphology of tumor as assessed microscopically on a scale of 1 to 3, reflecting increasing differences in appearance compared with normal epithelium. The final tumor grade is derived from these three scores. With the increased utilization of digital pathology, grading can now be carried out on digitized histopathological images, which yields similar agreement as compared to assessment via light microscopy^3^. Increasingly, the pathologists can also be assisted by artificial intelligence (AI)-based systems in routine practice^4–7^.

The most commonly applied AI technology for analysis of medical images are so-called deep neural networks (deep learning; DL). Deep-learning architectures are composed of connected neurons that receive an input image and perform a series of operations on the learnable network parameters to accomplish a learning task, such as classification, regression and segmentation. They have led to unprecedented success in several areas of computer vision^8–11^ to more recent advancements in digital pathology^12–16^. More specific for breast cancer grading, automated mitosis detection was one of the first applications of DL^17,18^, demonstrating its clinical and prognostic value^19–21^. Gland segmentation was formulated as a series of nuclei and gland detection, as well as a segmentation task^22,23^, with extensions to clinical risk categories^24^ through the use of DL.

Unlike gland formation and mitotic count, nuclear pleomorphism does not have the same quantitative nature in its definition. As a result, nuclear pleomorphism scoring is the least reproducible of the three grading components, which limits its utility^25–27^. Owing to the qualitative nature of nuclear pleomorphism scoring and the difficulty in forming a reference standard, the existing works in this field have been limited. In multiple works^28–31^, traditional ML techniques were applied on hand-crafted features for a standard three-category classification of nuclear pleomorphism on patch-level instances. Another work^32^ employed a convolutional neural network to learn relevant features for the binary classification of severe (score 3) pleomorphism, and sparse representations of feature embeddings^33^ with an extension to active learning^34^ were formulated for the patch-level three-way classification of nuclear pleomorphism. Contrary to the existing works, our approach utilizes a reference standard from the collective knowledge of 10 pathologists, the largest in any related work, in an effort to reduce the inter-observer variability. Another contribution of our work is the reformulation of the discrete three-category classification of nuclear pleomorphism scoring into a regression task over the full spectrum of nuclear pleomorphism. Finally, we perform qualitative and quantitative analyses of our approach on selected regions of interest and on an external set of whole-slide images to compare with the panels of 10 and 4 pathologists, respectively.

Supervised DL requires reference standard labels associated with the images for neural network training. Acquiring such labels is a non-trivial task for applications such as pleomorphism scoring, with discrete classes and observer variability. Translating the scores of multiple observers into a reference label (e.g., based on majority, or consensus) disregards valuable information, which is present in the spread of the scores of individual experts. In this work, we therefore formulate a novel DL approach to nuclear pleomorphism scoring by considering it as a continuous score, rather than classifying it into three discrete categories. We re-formulate the original three-category classification into a full spectrum of continuous values from the lowest pleomorphism score to the most severe.

**Fig. 1:**
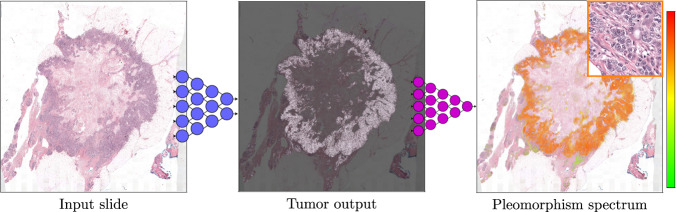
Overview of the AI algorithm scoring nuclear pleomorphism spectrum. An input whole-slide image is processed through the epithelial cell detection network to detect the tumor regions. Subsequently, the deep regression network scores nuclear pleomorphism on the tumor regions, score 1 is denoted by green, score 2 by yellow and score 3 by red color. Since the deep regression network outputs a spectrum of values, ranging from 1 to 3, the pleomorphism spectrum reflects the values in between the traditional three categories, as well. In this example, the AI algorithm scored high pleomorphism for the tumor regions in the input slide, evident by presence of dark orange and red colors from the pleomorphism spectrum in the detected tumor region.

Our approach mainly consists of two parts. The first part is an epithelial cell detection network developed previously^35^ (see “Methods” section for details). This step is intended to limit the analysis to the diagnostically relevant regions (i.e., invasive tumor) within a whole-slide image. In the second step, a deep regression network predicts the continuous nuclear pleomorphism score on the tumor regions. Training this network does not require detailed manual annotations by pathologists, which is one of the key limiting factors within computational pathology. This work marks the first end-to-end fully automated nuclear pleomorphism scoring in breast cancer using DL. Moreover, we are translating a discrete classification into a continuous regression problem to preserve valuable observer input, which is not applied previously. The overview of our approach outlining this two-stage process of nuclear pleomorphism scoring is presented in Fig. 1. We refer to our approach as “AI algorithm” or "AI” throughout the rest of the paper.

In order to develop and validate our approach, we carried out two separate studies, resulting in two separate data sets (see Table 1 for details). The first study, referred to as *ROI-study*, consisted of 125 tumor regions of interest (tumor ROIs) and an additional 79 “normal appearing” epithelial regions of interest (normal ROIs) from 39 whole-slide images, carefully selected to include a wide variety of tumor morphology in breast cancer histopathology. We invited a panel of 10 pathologists from 6 countries to score nuclear pleomorphism on the tumor ROIs, each visually paired with a normal ROI from the same patient for the pathologists to use as reference for scoring the degree of pleomorphism. We used the results of the ROI-study to train our AI algorithm as well as to evaluate its performance compared to the panel of pathologists. In our automated approach, we used the averaged nuclear pleomorphism scores of the 10 pathologists as reference scores, representing their collective knowledge rather than forcing a discrete majority score. The AI algorithm was trained on a wide range of tumor morphology, leveraging the reference scores to establish the concept of nuclear pleomorphism as a full spectrum of tumor morphology change.

**Table 1:** Overview of the number of regions of interests (ROIs), whole-slide images (WSIs), and pathologists involved in the two reader studies of this work, namely the ROI study and the slide study.

	Tumor ROIs	Normal ROIs	WSIs	Pathologists	Target
ROI study	*n* = 125	*n* = 79	*n* = 39	*n* = 10	ROI-level pleomorphism
Slide study	–	–	*n* = 118	*n* = 4	Slide-level pleomorphism

Target indicates what pathologists had to score in the two reader studies, namely a single-pleomorphism score for each ROI in the ROI study, and a single-pleomorphism score for each slide in the slide study.

In the second study, referred to as the *Slide-study*, 118 whole-slide images of breast cancer resection tissue sections were used for evaluation. The distribution of the slides in this study resembled the distribution of the cases in routine clinical practice. We invited four pathologists to score nuclear pleomorphism of the slides, which were then compared to our AI algorithm for whole-slide-level evaluation. The ROI- and Slide-study, as well as the procedure to train AI algorithms used in this study are outlined in more detail in the “Methods” section.

## Results

In Table 2, we provide an overview of the data used for training and validation purposes in this work. The training set came from 52 ROIs selected from the 16 slides in the ROI-study. The best model was selected based on the performance (i.e., smallest loss value, see “Methods” section for details) on the validation patches from the 28 ROIs within 10 slides. We considered the spread of the reference scores of the pathologists for the selection of training and validation slides to expose the AI algorithm to as much variety as possible. The remaining 45 ROIs from the 13 slides were used for evaluation. On the other hand, the entire set of the 118 whole-slide images in the slide-study was exclusively used for evaluation.

**Table 2:** Overview of the number of patches, regions of interests (ROIs) and whole-slide images (WSIs) used for training and validation purposes in this work.

	Patch-level training	Patch-level validation	ROI-level validation	WSI-level validation
ROIs	*n* = 52	*n* = 28	*n* = 45	–
WSIs	*n* = 16	*n* = 10	*n* = 13	*n* = 118
Patches	*n* = 2400/epoch	*n* = 6000/epoch	–	–

### Prediction performance of the AI algorithm

**Fig. 2:**
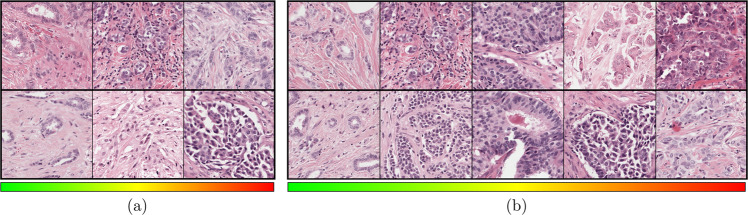
Nuclear pleomorphism predictions quantized into different numbers of categories, sorted from low pleomorphism to high in each category, from left to right. In **a**, patches were quantized into three categories, which was in line with the three-category classification in routine clinical practice, whereas in **b**, the patches were quantized into five categories to demonstrate the continuity of the nuclear pleomorphism spectrum from the predictions of the AI algorithm.

In this section, we analyzed the predictions of the AI algorithm on fixed-sized patches of 512 × 512 pixels randomly cropped at 20× magnification from 45 ROIs out of the 13 evaluation slides in our ROI data set. The output of our AI algorithm was a continuous numerical value ranging between 1 to 3, corresponding to the increasing severity of the nuclear pleomorphism. For patch-level quantitative analysis of the prediction performance of the deep regression network, we sampled four independent sets of 1000 patches from the validation set. Our approached achieved a MAE of 0.262 ± 0.004, MSE of 0.111 ± 0.002 and *EV* score of 0.756 ± 0.009. The regression errors (MAE and MSE) from the AI algorithm were small with respect to the prediction window [1 to 3] and the EV score was high. For patch-level qualitative analysis, we demonstrated the granularity of our automated approach by quantizing the predictions into the three categories as per the guidelines, as well as five additional categories. In Fig. 2, we present example patches from the test set for each category, sorted by the quantized predictions of the AI algorithm, with each patch having a higher categorical score than the one preceding it. While the nuclei in the leftmost patches were closest in appearance to healthy epithelium, the degree of nuclear pleomorphism became gradually more severe with each patch to the right.

**Fig. 3:**
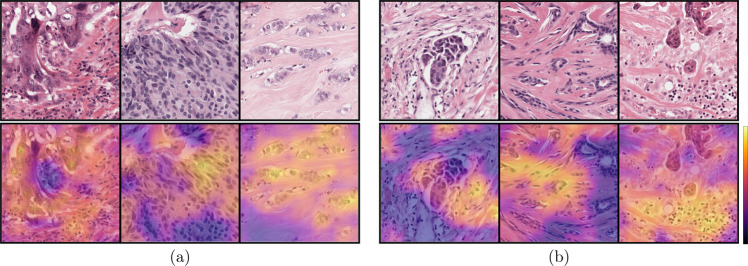
Grad-CAM saliency outputs of six patches from the evaluation set in the ROI-study. The predictions were close to the reference scores (**a**) when the AI algorithm focused on the cellular structures and it failed in few cases (**b**) focusing on other tissue parts.

We selected the gradient-weighted class activation mapping (Grad-CAM)^36^ method to qualitatively investigate the visual cues that the AI algorithm learned to score pleomorphism. Through this method, we could highlight the areas in a high-resolution input image patch to inspect whether the prediction was based on nuclear morphology. In Fig. 3, we present several example patches from the ROI test set, visualizing the salient areas determined by the deep regression network. The patches with predictions close to the reference scores showed strong activation around nuclei. Similarly, when we inspected the patches where the AI algorithm failed, we observed that it overlooked the nuclear structures and incorporated data from other tissue areas. Our qualitative experiments in this section indicated that when looking at high-resolution local predictions at patch level the AI algorithm predicted similar scores with the pathologists when it focused on the nuclei, failing when focusing on other areas, such as stroma, in very rare occasions. In ROI- and slide-level experiments, multiple overlapping patches with small displacements were scored to mitigate the rare occurrence of this problem, resulting in predictions based on the nuclear structures. This Grad-CAM based visualization technique can be extended to ROI- or WSI-level via a sliding window approach based on the analysis of high-resolution patches.

### Quantitative comparison of the AI algorithm

**Fig. 4:**
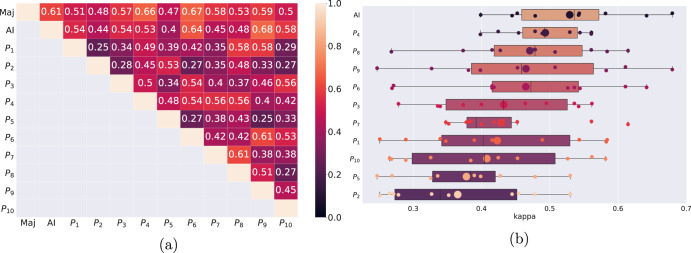
Quadratic kappa scores of the pathologists as well as the AI algorithm on the evaluation set in the ROI-study (45 ROIs). In **a**, pairwise kappa scores of the AI algorithm and the pathologists are compared where the best pairwise kappa score was achieved between the AI algorithm and *P*_9_. The majority scores, denoted by Maj, had the third best kappa score with the AI algorithm, behind *P*_6_ and *P*_4_. The pairwise kappa metrics, sorted by the average pairwise scores, **b** shows that the AI algorithm has, on average, the highest agreement out of any pathologists. It also has the highest pairwise kappa score of 0.68 with *P*_9_. In each row, small dots correspond to the kappa scores with the other pathologists/AI algorithm and the large dot denotes the average kappa score of that particular pathologist/AI algorithm with the others.

Ten pathologists scored nuclear pleomorphism into one of the three categories, 1 to 3, in the ROI-study, which was curated to contain as much as possible a uniform degree of pleomorphism. The first quantitative comparison between the AI algorithm and the pathologists used the evaluation set of this study, consisting of 45 ROIs from 13 slides. We quantized the predictions of the AI algorithm into the three categories to compare to the scores of the individual pathologists, as well as to the majority vote of their scores (using the provided confidence scores in the case of ties). We present the kappa scores quantifying the agreement between the pathologists and the AI algorithm in Fig. 4. In this comparison, the scores of a pathologist were not part of the majority voting in their comparison to the majority scores. The AI algorithm had a kappa score of 0.61 with the majority scores (denoted as “Maj” in Fig. 4a), performing better than 8 out of the 10 pathologists. It only trailed behind the two pathologists, *P*_6_ and *P*_4_, whose kappa with the majority scores were 0.67 and 0.66, respectively. Moreover, the AI algorithm had the highest average kappa score of 0.53 in pairwise comparisons, followed by *P*_4_ with a kappa score of 0.49.

### Towards application in clinical practice: nuclear pleomorphism scoring on whole-slide images

**Fig. 5:**
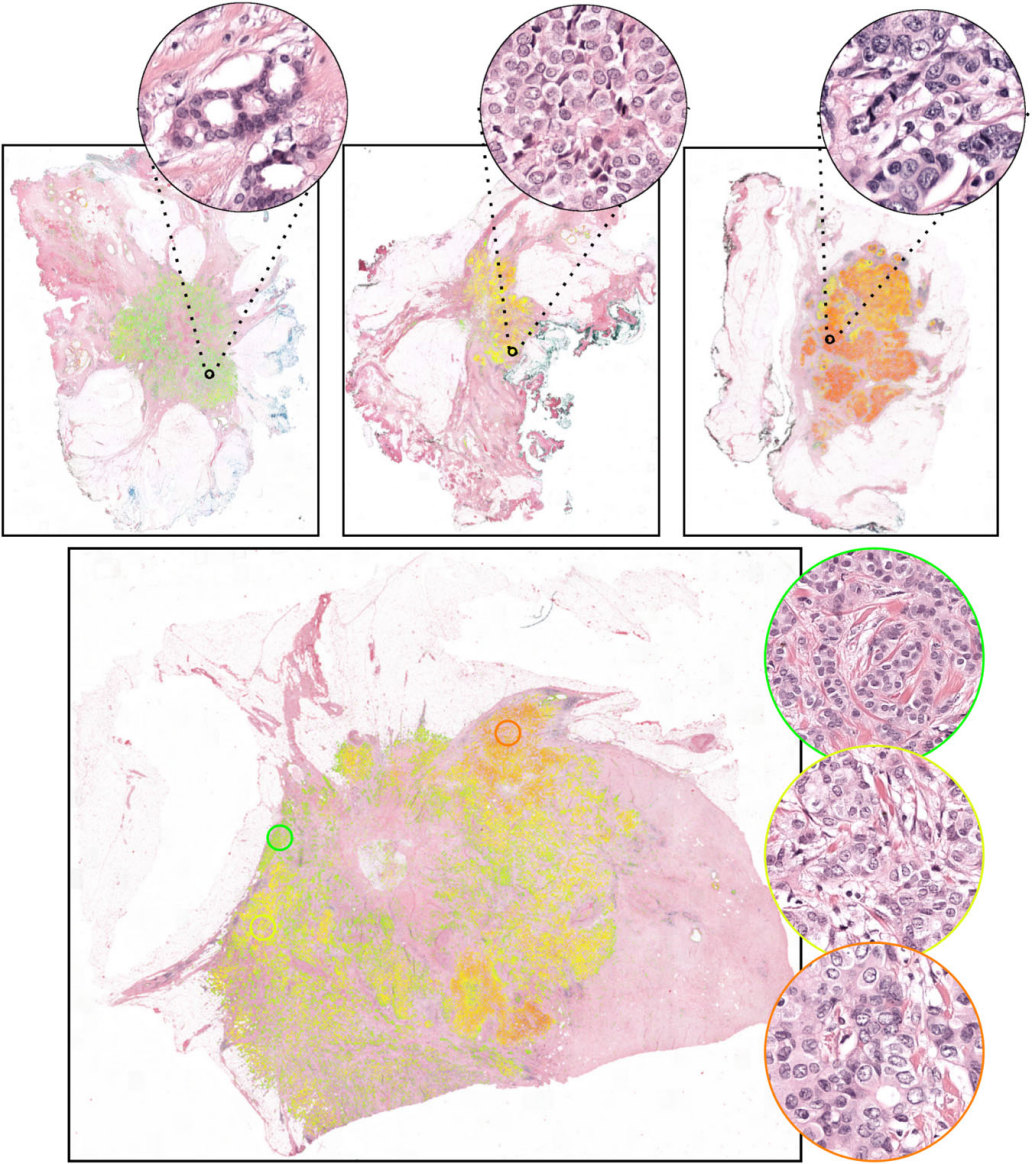
Nuclear pleomorphism spectrum of the AI algorithm on 4 of the 118 evaluation slides in the slide-study. The color spectrum from green to yellow to red denotes increasing nuclear pleomorphism severity. The deformation in nuclear morphology with the increasing pleomorphism scores demonstrated the capability of the AI algorithm capturing the concept of nuclear pleomorphism. The four pathologists scored (1, 2, 1, 1), (1, 2, 1, 2) and (2, 3, 2, 3) for the top three slides from left to right, with predictions displaying mostly uniform tumor morphology across the slides. All pathologists scored 2 for the slide at the bottom, which contained a larger range of tumor morphology, evident from the predictions of our approach.

In the slide-study, our experimental setup was in line with the real-world clinical setting, where a single score is given to an entire slide by a pathologist. The slide-level evaluation between the AI algorithm and the four pathologists, *P*_*i*_, *P*_*ii*_, *P*_*iii*_, *P*_*iv*_, were on the full set of 118 whole-slide images, which were scored into one of the three categories. In contrast to the ROI-level study in which nuclear pleomorphism in an ROI was homogeneous, whole-slide images in this study were heterogeneous with a larger variety of nuclear morphology. We present the visual pleomorphism spectrum of the (non-quantized) predictions of the AI algorithm on four example slides in Fig. 5. Qualitative analysis of the visual outputs on whole-slide images demonstrates the use-case of the AI algorithm as a time-saving, practical tool for nuclear pleomorphism scoring.

**Fig. 6:**
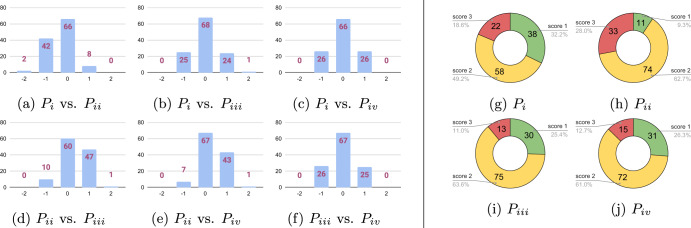
Comparison of pleomorphism scores in the slide-study. The comparison of the pleomorphism scores of the four pathologists **a**–**f** as well as the score distribution of the pathologists **g**–**j** in the slide-study display the scoring patterns of the pathologists on the 118 whole-slide images in the slide-study. The largest differences were observed between *P*_*ii*_ and other pathologists, with *P*_*ii*_ assigning significantly lower number of score 1s and much higher number of score 3s compared to the other three pathologists.

**Fig. 7:**
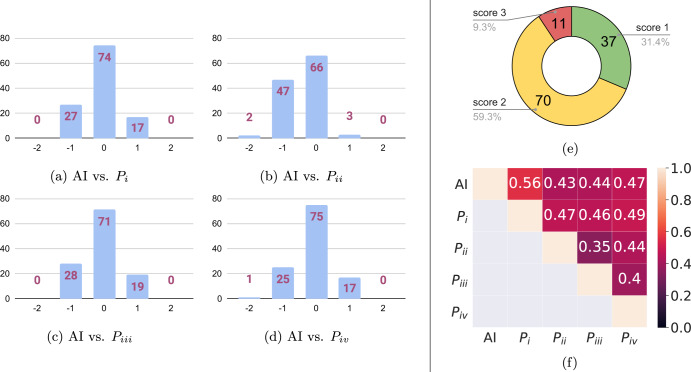
Performance evaluation of the AI algorithm on the 118 whole-slide images in the slide-study. For comparison purposes, the scores of the AI algorithm were quantized into three categories. The score differences of AI algorithm from the four pathologists are presented through **a** to **d**, while its score distribution is shown in **e**. The AI algorithm and the first pathologist, *P*_*i*_, had the highest agreement (**f**) with AI being only second to *P*_*i*_ on average pairwise kappa scores.

For the quantitative analysis of the AI algorithm compared to the pathologists, we quantized the pleomorphism predictions of the slides into three categories. The score differences illustrated in Fig. 7(a) through (d) reveal that the AI algorithm achieved a nuclear pleomorphism score equal to pathologists *P*_*i*_, *P*_*ii*_, *P*_*iii*_, *P*_*iv*_ in 74(%63), 66(%56), 71(%60) and 75(%64) slides, respectively. Moreover, it had a score difference of ±1 on the rest of the slides, except for three slides; two of which were with *P*_*ii*_ and the third one was with *P*_*iv*_. For these cases, the scores of the four pathologists were (1, 3, 2, 1) for the first slide, (2, 3, 2, 2) and (2, 2, 2, 3), for the second and the third slide, respectively. The quantized pleomorphism score of the AI algorithm was 1 for these slides. In addition, it matched the scores of *P*_*iv*_ in 75 slides, the highest in any pairwise comparison (see Fig. 6 for pairwise comparison of the pathologists). The quantized score distribution of the AI algorithm over all the slides in the study is given in Fig. 7e. Finally, we present the pairwise quadratic kappa scores between AI and the pathologists in Fig. 7f. The highest pairwise kappa score of 0.56 was achieved between the AI algorithm and *P*_*i*_. AI had pairwise kappa scores of 0.43, 0.44 and 0.47 with the rest of the pathologists, *P*_*ii*_, *P*_*iii*_, and *P*_*iv*_, respectively. On average, the pairwise kappa score for the AI algorithm was 0.475 and it was only second to *P*_*i*_ with 0.497. The slide-level results highlight the top-level performance of our approach, and they are consistent with the previous results in ROI-level experiments, with AI consistently ranking high in agreement with the best performing pathologists.

**Fig. 8:**
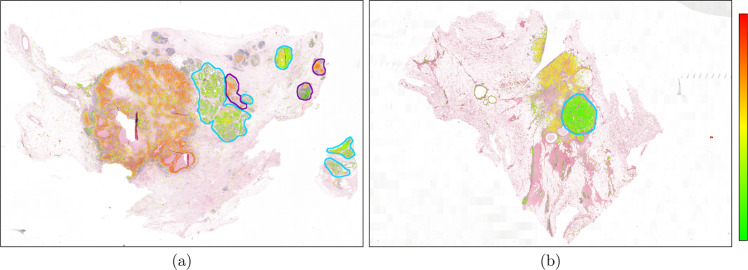
Examples of sub-optimal behaviour of the proposed algorithm. Non-invasive malignant and benign tissue, outlined in burgundy and cyan colors, respectively, could be mistaken as invasive tumor by the epithelial cell detection network. This sub-optimal behavior altered the quantized pleomorphism scores of two slides by one in the test set. The slide on the left (**a**) had a highly pleomorphic tumor region, which suggested a quantized pleomorphism score of 3, but the low scores from the benign areas pulled down the overall score to 2. All four pathologists scored 3 for the slide. Similarly, the pleomorphism score of the slide on the right (**b**) should have been 2, evident by the tumor region in yellow color, but it was quantized to 1 due to the low scores from the large benign area. All four pathologists scored 2 for the slide.

The AI algorithm was fully automated as no human or expert fine-tuning took place during training or inference. In few cases, false tumor predictions by the epithelial cell detection network resulted in scoring pleomorphism in non-invasive areas. There were two such instances in our slide-level experiment where the AI performance was compromised, as displayed in Fig. 8. The low degree of pleomorphism in benign areas caused the overall scores of both slides to go down by one. Excluding the scores in the benign areas from the two slides would further improve the slide-level kappa scores of the AI algorithm; 0.59, 0.45, 0.46, 0.50 from 0.56, 0.43, 0.44, 0.47 with pathologists *P*_*i*_, *P*_*ii*_, *P*_*iii*_, *P*_*iv*_, respectively. In rare cases, such as the visually outlined areas in Fig. 8a, in situ carcinoma was also scored. In this case, the scores in these areas did not alter the overall score due to having similar degree of nuclear pleomorphism with the invasive tumor or being too small to make an impact. We argue that re-training the epithelial cell detection network with a more diverse set of benign examples, and employing a multi-resolution segmentation approach with a larger field of view, such as ref. ^37^, could resolve these issues.

## Discussion

In this work, we reported a fully automated deep-learning method for scoring nuclear pleomorphism in breast cancer through reformulating the standard classification as a spectrum of the extend of the changes in tumor morphology. Our AI algorithm was composed of two stages; an epithelial cell detection network was used to locate the invasive tumor in whole-slide images, and subsequently, a deep regression network scored nuclear pleomorphism on the tumor, considering the nuclear pleomorphism as a spectrum.

In our experiments, we quantized the patch-level predictions of the AI algorithm into arbitrary numbers of categories to showcase its flexibility of demonstrating pleomorphism continuity in greater granularity than the traditional three-category classification. In few patch-level classification experiments, we noticed that the AI algorithm did not focus on areas with tumor cells, thus failing to capture the tumor pleomorphism. Overlapping patch-level predictions for ROI- and slide-level inference mitigated this rare-occurring problem through pooling. We demonstrated that our automated approach could learn to score pleomorphism from tumor only, not requiring normal epithelium for comparison. In our ROI-level experiments, the AI algorithm achieved a higher agreement with the majority scores than 8 out of the 10 pathologists, and it had the best overall spread of pairwise kappa scores among all pathologists. The slide-level experiments suggested a similar outcome in which the AI algorithm had the second-best overall spread of pairwise kappa scores among four pathologists. The kappa scores of the best performing pathologist was the highest with the AI algorithm compared to the other three participating pathologists. In few slide-level predictions, the epithelial cell detection network had false tumor predictions resulting in scoring on those regions. This minor issue could be resolved by employing a more powerful tumor segmentation approach with a larger field of view, such as^37^. Overall, we demonstrated that the AI algorithm consistently achieved top-level performance similar to the best performing pathologists throughout our quantitative experiments in ROI- and slide-study. The results proved that the careful curation of our training data set covering a large range of tumor morphology to account for the nuclear pleomorphism spectrum as well as the data augmentations to make the AI algorithm resilient against the variations in the visual appearances of the H&E slides due to different sources were sufficient for top pathologist-level performance. We argue that for domains with limited visual space, such as the extent of the abnormalities of tumor nuclei in breast cancer, curated data sets representative of the label space with domain-specific data augmentations enable DL solutions to achieve high performance.

In routine clinical practice, nuclear pleomorphism scoring of whole-slide images from breast cancer patients requires a great amount of time for pathologists due to the investigation of multiple tumor regions at a high (20× − 40×) magnification and the comparison to that of normal breast epithelium. Our AI algorithm provides pleomorphism scores for tumor regions in whole-slide images, making it an automated stand-alone tool for breast cancer grading. Moreover, it can aid pathologists by visualizing the pleomorphism scores on the images at a glance; therefore, improving the pathologist efficiency in daily practice. Our approach makes it possible to investigate the importance of the nuclear pleomorphism score distribution on images at different classification ranges than the standard three categories for the prognostic analysis of the patients. Even beyond, the evolution of the tumors can be visualized from the spatial distribution of the continuous nuclear pleomorphism scores.

Our study has some limitations. First, we have restricted the analysis and the validation to one single slide per patient, whereas in clinical practice, pathologists score pleomorphism based on multiple slides per patient, usually by mentally averaging scores across slides. In our future work, we are planning to extend the validation of the proposed method to multiple available slides per patient. However, the proposed method will be directly applicable to the new setting, as the automated pleomorphism score is computed by averaging predictions across several regions within a single slide, an operation that can be naturally extended to multiple slides without changing the core algorithm. Second, in the current study we solely address the potential impact of this algorithm as a computer-aided diagnosis (CAD) tool, aiming at supporting pathologists in clinical diagnostics. However, in our future work, we will study the potential value of the automated pleomorphism score as a prognostic digital biomarker, focusing on both the analysis of prognostic impact of different cut-off values, as well as on the information contained in the spatial distribution of nuclear pleomorphism on whole-slide images for overall survival of breast cancer patients.

## Methods

In this section, we describe the data used in this study to build the ROI-study data set and the Slide-study data set, as well as details on the deep-learning algorithm for automated nuclear pleomorphism scoring. All data were collected from the Radboud University Medical Center (Nijmegen, Netherlands). The local Institutional Review Board waived the need for approval to collect and use data in this study (#2015-1637).

### ROI-study data set

We collected H&E stained whole-slide images of breast cancer resections with a total number of 39 slides from two cohorts, which we refer to as cohort A and cohort B. The slides in cohort A were scanned on a 3DHistech P1000 scanner at 0.25 μm/pixel, and the slides in cohort B were scanned on a 3DHistech Pannoramic 250 Flash II scanner at the same spatial resolution of 0.25 μm/pixel. From cohort A; we made a balanced selection of cases with respect to the nuclear pleomorphism scores. Invasive carcinoma of No Special Type (NST) was the most prevalent tumor type, seen in 24 out of the 31 slides, resembling the distribution of breast cancer patients in clinical practice. From the cohort B; we included 8 additional cases that covered different histological subtypes with severely aberrant morphology from a triple negative breast cancer cohort. As a result, pleomorphism of the tumor with score 3 in cohort A was, on average, less severe than the tumor with score 3 in cohort B. The full distribution of the carcinoma types in the selected cases is provided in Table 3. Overall, the slides were selected to cover the entire range of nuclear pleomorphism from a large spectrum of tumor morphology in breast cancer pathology.

**Table 3:** Distribution of the tumor types in the ROI- and slide-study.

Invasive carcinoma type	ROI-study		Slide-study
	Slides	ROIs	Slides
No special type (NST)	26	83	91
Lobular	6	21	22
Metaplastic carcinoma	3	8	–
Invasive micropapillary	1	4	2
Mucinous	1	3	–
Tubular	1	3	2
Malignant adenomyoepithelioma	1	3	–
Cribriform	–	–	1
Total	39	125	118

In clinical practice, normal epithelium in a whole-slide image, when present, is a useful reference point for pathologists to determine the degree of pleomorphism of the tumor. In the ROI-study, we presented pairs of ROIs from tumor and normal epithelium to the pathologists for nuclear pleomorphism scoring similar to the routine practice. We manually selected one or more tumor ROIs from each slide ensuring pleomorphism homogeneity within each ROI. Overall, we selected 125 such regions from 39 slides. Additionally, we manually selected at least one ROI from normal appearing epithelium (normal ROI). As few slides contained little to no large enough areas with normal epithelium, it was not possible to select at least one normal ROI from every slide. In total, there were 59 normal ROIs that we could select from the set of slides. Ten out of the 39 slides did not have large enough region with normal cells. Therefore, for the whole-slide images without normal epithelium, we retrieved 10 additional slides from the same patient to acquire at least one normal ROI. As a result, the total number of selected normal ROIs increased to 79 with the selection of 20 normal ROIs from the additional set of slides. Following this selection procedure, each ROI was cropped around their center point to a square area of around 0.38 mm^2^ at 0.25 μm/pixel. Subsequently, a tumor ROI was paired with a normal ROI from the same patient to form a query pair. This process was repeated for all 125 tumor ROIs in the data set. It has to be noted that each query had a unique tumor ROI, but a normal ROI could be paired with more than one tumor ROI due to the smaller number of normal ROIs compared to tumor ROIs, 79 vs. 125.

**Fig. 9:**
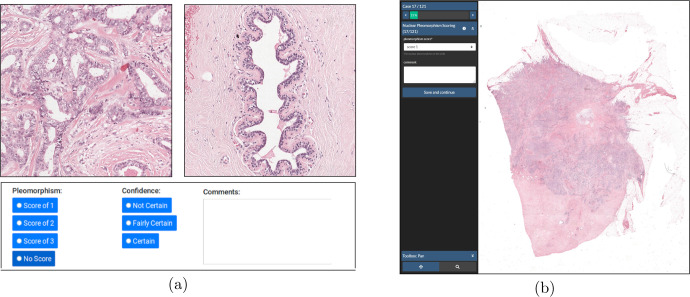
Web platforms for the ROI- and slide-study. An example query from the web platform of the ROI-study (**a**) included a pair of tumor and normal ROIs from the same patient. Ten pathologists were invited to score nuclear pleomorphism and to provide confidence scores for such 125 queries from 39 whole-slide images. An example from the slide-study (**b**) included a whole-slide image, which could be viewed in multiple magnifications. Four pathologists were invited to score nuclear pleomorphism on 121 whole-slide images.

**Fig. 10:**
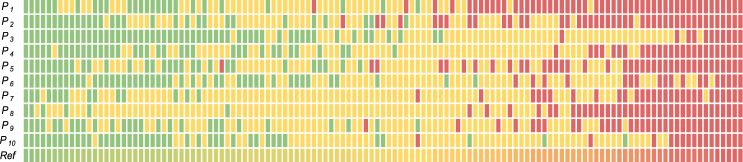
Distribution of the nuclear pleomorphism scores of the pathologists and of the average (reference) scores of the pathologists in the ROI-study. The rows correspond to the pathologists, ${\{{P}_{g}\}}_{g = 1}^{10}$, except for the last row that corresponds to the average scores of the pathologists, which we used as reference scores in our learning setup. Each column is one of the 125 queries in the ROI-study. The colors green, yellow and red correspond to the scores of 1, 2 and 3 of the pathologists, respectively. The queries are sorted by the average scores, from left to right in increasing order, denoted by the color hues of green, yellow and red, corresponding to the increasing severity of the pleomorphism.

We built a web-based nuclear pleomorphism platform consisting of the queries to display the ROIs, allowing the pathologists to score them through a user interface. Each query was followed by the questions; nuclear pleomorphism score of the tumor into one of the three categories; 1, 2, or 3, and an optional field for confidence with the scoring; not certain, fairly certain, certain, as well as an additional text field for comments about the queried tumor and normal ROIs. An example is provided in Fig. 9a. When an ROI was selected, the full resolution of the ROI was displayed with a total size of 2560 × 2560 pixels. We invited 10 pathologists from 6 countries with varying levels of expertize in breast pathology to participate in this study. As a result, each tumor ROI was scored for nuclear pleomorphism 10 times. For a tumor ROI, we aggregated the pleomorphism scores of the pathologists by average pooling. We illustrate the reference scores as well as the pleomorphism scores of the individual pathologists for all 125 queries in Fig. 10. Throughout our experiments, we trained and validated the AI algorithm on a large subset of this data set, using the reference scores as reference standard. The rest of this data set was used to evaluate the ROI-level performance of the AI algorithm compared to the pathologists. A more detailed breakdown of the training, validation and evaluation subsets is provided in the experimental settings section.

### Slide-study data set

We collected an additional set of H&E stained whole-slide images from cohort B, with a total number of 118 slides. The slides selected for this study did not have any overlap with ROI-study, as the patients in each study were unique. The selection criteria for the slides followed a similar approach to ROI-study in that we collected a large variety of slides with diverse tumor morphology. We referred to the available clinical data of the patients for our selection to approximate the distribution in the clinical practice. As a result, NST was the most prevalent carcinoma type with 71 slides, followed by Invasive Lobular Carcinoma (ILC) with 14 slides. The full distribution of the carcinoma types in the slide-study is presented in Table 3.

A web-based reader study based grand-challenge.org (www.grand-challenge.org/reader-studies) was built for scoring nuclear pleomorphism on the 118 slides. The application could display multi-resolution whole-slide images to enable pathologists to freely navigate the slides with zoom-in and zoom-out capabilities. In this study, we asked pathologists to score nuclear pleomorphism of whole-slide images into one of the three categories; 1, 2, or 3, and we provided them an optional comment field for each queried slide, as displayed in Fig. 9b. We invited 3 of the 10 pathologists who participated in the ROI-study, to also score nuclear pleomorphism in the slide-study. Moreover, we invited an additional pathologist who did not participate in the ROI-study, bringing the total number of pathologists to four. Detailed breakdown of the score distributions of the pathologists, individually, and compared to each other is displayed in Fig. 6. In our experiments, we used the whole-slide images and the pleomorphism scores of the pathologists only to evaluate the performance of the AI algorithm, as opposed to the ROI-study in which training and validation of the AI algorithm took place.

### Automated nuclear pleomorphism scoring

Our approach for learning nuclear pleomorphism scoring followed a two-stage methodology (see Fig. 1). The first stage consisted of the detection of tumor and normal cells using an epithelial cell detection network developed in-house and previously presented^35^. In brief, a RetinaNet detection model^38^ was trained with image patches of 256 × 256 pixels at 40× magnification from breast cancer whole-slide images stained with H&E. Slides contained manual annotations of tumor and normal epithelial cells in the form of point annotations, which were converted to fixed-size bounding boxes for training purpose. At test time, the trained RetinaNet model predicts bounding boxes centered at the location of detected epithelial cells at slide level, and labels each prediction as containing a normal or a tumor epithelial cell.

**Fig. 11:**
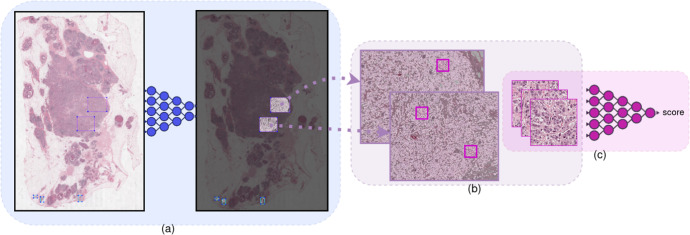
Proposed methodology for learning nuclear pleomorphism scoring by tumor morphology as a spectrum. Patches were sampled from areas with high nuclear composition (**a**, **b**) using epithelial cell detection network. The weights of the epithelial cell detection network (**a**) were kept frozen during the training of the deep regression network (**c**) for learning pleomorphism spectrum.

The second stage contained the training of a deep regression network on tumor to learn nuclear pleomorphism scoring as a spectrum instead of the traditional three-category classification. An illustration of the proposed methodology is presented in Fig. 11. During training of the regression network, patch sampling strategy involved locating the areas with high nuclear composition within the selected ROIs in the ROI-study by the epithelial cell RetinaNet model. In this work, we utilized this pre-trained network, keeping its weights frozen during training and inference. This step was particularly important in that it enabled learning from nuclear features rather than from other factors in the tissue. For inference, the epithelial cell detection network pre-processed whole-slide images to locate invasive tumor regions for the deep regression network to score nuclear pleomorphism.

Nuclear pleomorphism is traditionally scored into one of the three categories from 1 to 3, indicating the degree of abnormalities in the tumor appearance. Given that nuclear pleomorphism reflects the extent of abnormalities in the appearance of tumor nuclei, we considered a more generalized version of the categorical classification in the form of a continuous spectrum of nuclear pleomorphism. The granularity in the pleomorphism spectrum came from the averaged collective knowledge of 10 pathologists. This realization transformed the three-category classification problem into a regression task. Therefore, the second stage in our methodology was comprised of a Densenet^39^ based deep regression network, which was trained on the tumor patches using the average scores of the pathologists as reference standard. The network architecture consisted of “dense blocks” in which the input of a layer was the concatenation of the output of the previous block and the inputs of the layers preceding it. Another distinguishing feature was the “transition layers”, which helped to regulate the number of channels in the network. In comparison, a traditional CNN is only composed of consecutive layers, in which an input of a layer is the output of the previous layer.

#### Training

A training iteration of our Densenet based deep regression network was as follows. A training patch sampled from the tumor ROI was fed through the deep regression network to regress its pleomorphism score. A *smooth*_*L*1_ loss function^40^ was computed from the prediction with respect to the reference score. Finally, the gradient of the loss function was backpropagated through the network to improve the prediction performance in the next iteration by updating the network parameters. This training iteration was done on a batch of multiple patches in the training set. We illustrate one such training iteration in Fig. 11 through (a) to (c). Tens of thousands of patches with high nuclear composition and the granularity of the reference scores enabled the AI algorithm to learn nuclear pleomorphism as a spectrum.

We trained the network from scratch using the Adam optimizer with an initial learning rate of 1*e* − 4, which we decreased by 30% during training when no improvements were observed on the validation set for ten epochs. Furthermore, we paired Adam with the decoupled weight decay regularization technique proposed in this paper^41^. Each training patch was 512 × 512 pixels at 0.5 μm/pixel spacing, sampled from the tumor ROIs in the training set. We used a batch size of 12 patches in each training iteration, and 200 training and 500 validation iterations per epoch. Training continued until convergence, i.e., no improvement on the validation loss in 10 consecutive epochs. Spatial data augmentation operations such as horizontal and vertical flips as well as 90^∘^, 180^∘^, 270^∘^ rotations were applied to both training and validation patches. In order to make the AI algorithm robust against the variations in color and staining, we applied a large range of color and stain augmentations on the training patches, as discussed in^42^. The training patches were also augmented with blurring techniques to make the network more robust against out-of-focus regions. Nuclear composition in the training and validation patches was maximized through sampling from the nuclear density maps of the ROIs, obtained by applying Gaussian filters on the output of the epithelial cell detection network. The patch-level performance of the deep regression network was quantified using several regression metrics; mean absolute error (MAE), mean squared error (MSE) and explained variance (EV) score. The continuous-valued predictions of the AI algorithm were quantized into arbitrary numbers of categories to demonstrate the continuity of pleomorphism as well as for comparison against pathologist scores. Quantization of a continuous range of values into *p* categories was the process of partitioning the range into evenly sized *p* number of brackets, and representing each bracket with an integer value. To illustrate, the range [1, 3] was quantized into three categories as follows; any value in the range of [1, 1.66) corresponded to 1, [1.66, 2.33] corresponded to 2, and (2.33, 3] corresponded to 3. We evaluated the performance of the AI algorithm versus the pathologists and the pairwise performance of the pathologists, by Cohen’s quadratic kappa measure^43^.

#### Slide-level inference

We performed slide-level inference by first applying the epithelial cell detection network before the slide-level inference to ensure scoring only on invasive tumor. Successively, we processed the slide as small overlapping tiles, and translating the scores of the tiles into a single slide-level pleomorphism score. Each tile was the same size as the input image patch with 512 × 512 pixels at 0.5 μm/pixel. In our experiments, we selected an overlap value of 448 pixels between each tile, horizontally and vertically. The AI algorithm scored a continuous pleomorphism value for each tile. As a result, a block of size 64 × 64 pixels received multiple scores from the predictions of overlapping tiles. The scores were aggregated by average pooling to assign the pleomorphism score of the block. Finally, the pleomorphism score of the slide was determined by the average pleomorphism score of its blocks.

### Reporting summary

Further information on research design is available in the Nature Research Reporting Summary linked to this article.

## Supplementary information

Reporting Summary

## Data Availability

We made the data set of *n* = 118 whole-slide images analyzed in the slide-study publicly available, together with a web-based platform based on grand-challenge.org, which implements the evaluation procedure and computes results as depicted in Fig. 7. Via this platform, the test slides can be downloaded, processed locally and predictions can be uploaded to grand-challenge.org to be evaluated by computing pairwise Kappa scores with the procedure implemented in this paper. This will enable researchers to test their models and compare their results with ours as well as with other participants. The data set can be downloaded from Zenodo (doi:10.5281/zenodo.7285896)^44^. Both images and evaluation platform can be accessed via this link: https://breastpleomorphism.grand-challenge.org/.

## Code availability

This project was developed based on private digital pathology data using both closed-source and open-source Python libraries. The main components of the code of this project are listed below: scripting language: Python v3.7; math/statistical operations: numpy v1.20 and pandas v1.2.4; deep learning: torch v1.9 and torchvision v0.10; visuals/plots: matplotlib v3.3.4 and seaborn v0.11 ; patch extraction: we used a Python package developed internally, which has been recently integrated into the public WholeSlideData software: https://github.com/DIAGNijmegen/pathology-whole-slide-data; color augmentation: we used a Python package developed internally, which has been recently integrated into the public StainLib software: https://github.com/sebastianffx/stainlib. Although the full source code of this project is not made publicly available, access to detail of its implementation can be granted under reasonable request.
